# The Serum Levels of the Soluble Factors sCD40L and CXCL1 Are Not Indicative of Endometriosis

**DOI:** 10.1155/2016/2857161

**Published:** 2016-04-17

**Authors:** Petra Pateisky, Dietmar Pils, Lorenz Kuessel, Ladislaus Szabo, Katharina Walch, Reinhard Obwegeser, René Wenzl, Iveta Yotova

**Affiliations:** Department of Obstetrics and Gynecology, Medical University of Vienna, Waehringer Guertel 18-20, 1090 Vienna, Austria

## Abstract

Endometriosis is a benign but troublesome gynecological condition, characterized by endometrial-like tissue outside the uterine cavity. Lately, the discovery and validation of noninvasive diagnostic biomarkers for endometriosis is one of the main priorities in the field. As the disease elicits a chronic inflammatory reaction, we focused our interest on two factors well known to be involved in inflammation and neoplastic processes, namely, soluble CD40 Ligand and CXCL1, and asked whether differences in the serum levels of sCD40L and CXCL1 in endometriosis patients versus controls can serve as noninvasive disease markers. A total of *n* = 60 women were included in the study, 31 endometriosis patients and 29 controls, and the serum levels of sCD40L and CXCL1 were measured by enzyme-linked immunosorbent assay. Overall, there were no statistically significant differences in the levels of expression of both sCD40L and CXCL1 between patients and controls. This study adds useful clinical data showing that the serum levels of the soluble factors sCD40L and CXCL1 are not associated with endometriosis and are not suitable as biomarkers for disease diagnosis. However, we found a trend toward lower levels of sCD40L in the deep infiltrating endometriosis subgroup making it a potentially interesting target worth further investigation.

## 1. Introduction

Endometriosis is a common gynecologic disorder that affects between 6 and 10% of women in their reproductive years [1]. It is already known that immunologic changes play a pivotal role in the development and progression of endometriosis [2]. An analysis of the peritoneal fluid of patients with endometriosis [3] showed differences in the expression pattern of chemokines, cytokines, and other proteins, compared to controls [4, 5], suggesting an altered microenvironment in the peritoneal cavity of endometriosis patients [6] that encouraged the development and persistence of endometriotic lesions [7, 8]. Based on this proinflammatory state of the ectopic lesion environment, endometriosis is often considered a condition that demonstrates patterns similar to that of a chronic systemic inflammatory disease [9]. Due to an increased cell proliferation rate, survival, and neovascularization in ectopic sites, the disease is often considered as a benign neoplastic condition. Moreover, some reports have shown that endometriosis is a risk factor for certain types of ovarian cancer [10, 11]. Changes in the tissue or the peritoneal cavity might be reflected by altered blood levels of several circulating proteins as well [12]. Yet, no single factor has been determined to serve as a reliable marker for the detection of the disease, not even the quite imprecise, partly advocated marker CA 125 [4, 13]. The combination of several differentially expressed factors appears to be the most promising approach in the search for a signature that would indicate a more precise suspicion and/or diagnosis of endometriosis [12, 14].

CD40 Ligand and CXCL1 (chemokine CXC motif ligand 1, synonym: GRO-*α*: growth-related protein-alpha) are proteins that play important roles in endothelial cell activation, the release of inflammatory cytokines, the regulation of apoptosis, and the regulation of angiogenesis and lymphocyte recruitment [15, 16]. Soluble CD40 Ligand is the soluble form of the CD40 Ligand, which is a type II transmembrane-bound protein. An increase of sCD40 Ligand in the blood is found in various autoimmune disorders, as well as in chronic inflammatory diseases [17, 18]. Recently, it has been shown that sCD40 Ligand levels are altered in the plasma of patients with PCOS [19, 20]. In a previous study of endometriosis patients, levels of CD40L and CD40 did not differ between patients and controls, but the sCD40 Ligand levels were not evaluated [21]. CXCL1 belongs to the *α*-subgroup of chemokines. It binds to the CXCR2 receptor and induces chemotaxis of neutrophils, lymphocyte migration, and angiogenesis via endothelial cell migration and proliferation [22, 23]. It has been shown that the expression pattern of certain CXC chemokines and their receptors is upregulated in endometriosis patients and in ovarian carcinomas [24]. Furthermore, data exists on higher concentrations of CXCL1 (GRO-*α*) in the peritoneal fluid of patients with endometriosis compared to patients without endometriosis [5], suggesting the involvement of this factor in the pathogenesis of endometriosis, possibly by influencing the microenvironment of the peritoneal cavity. However, the circulating levels of this chemokine in endometriosis have not yet been investigated.

In this study, we investigated the levels of sCD40 Ligand and CXCL1 in endometriosis patients and controls in order to search for possible differences in secretion levels. Furthermore, the potential of these proteins to serve as biomarkers either for detecting the disease or for identifying certain subgroups was evaluated.

## 2. Materials and Methods

### 2.1. Patient Population and Surgery

The present prospective cohort study was conducted at the tertiary referral certified Endometriosis Center of the Medical University of Vienna and was approved by the institutional ethics committee of the Medical University of Vienna (EK 545/2010). Between December 2010 and April 2012, sixty premenopausal women who were scheduled to undergo laparoscopic surgery were included in the study after their verbal and written informed consent prior to study inclusion.

Included were premenopausal women between 18 and 50 years of age who were scheduled to undergo surgery due to suspected endometriosis, pelvic pain of unknown reason, adnexal cysts, an infertility workup, or leiomyoma uteri. Women who had received hormonal treatment orally for at least one month prior to surgery and/or intramuscularly for at least three months, as well as patients with any malignant disease, acute inflammation or infection, and systemic autoimmune disorders, such as systematic lupus erythematosus or rheumatoid arthritis, were excluded from the study. Patient characteristics are shown in Table 1. Serum samples were obtained preoperatively from the patients directly, in a fasting state, on the day of surgery. In addition, all patients were asked to fill in a questionnaire in order to evaluate their pain symptoms (visual analogue scale (VAS): 0 = no pain; 10 = excessive pain), which, together with patients' detailed anamnesis sheet, resulted in a very well-characterized patient cohort.

The presence or absence of endometriosis was confirmed visually by laparoscopy and additional histopathological analysis. All surgeries were performed by the same group of experienced surgeons, who are part of the endometriosis core working group in our department. Endometriosis was classified according to the revised American Fertility Society Score (rAFS) [25] and in case of deep infiltrating endometriosis by the ENZIAN score [26]. Patients without evidence of endometriosis were classified as controls. The menstrual cycle phase was evaluated by histological analysis of endometrial biopsy obtained from every patient via diagnostic dilatation and curettage (D&C). In unclear situations, the specification was based on hormonal analysis.

### 2.2. Sample Preparation

The collected blood was centrifuged according to our standard protocol at 3000 rpm for 10 minutes at 4 degrees Celsius, 30 min to one hour after sampling. The serum was stored at −80 degrees for further processing. The serum sCD40 Ligand and CXCL1 (GRO*α*) concentrations were measured by enzyme-linked immunosorbent assay (ELISA) kits according to the manufacturers' protocols. All experiments were performed in duplicate. The sCD40L-serum levels were measured using the commercially available human sCD40L Instant ELISA (Cat. number BMS239INST human sCD40L, eBioscience). Concentrations were measured in ng/mL. The CXCL1 serum levels were measured using the commercially available human CXCL1/GRO alpha Quantikine ELISA Kit in concentrations as pg/mL (Cat. number DGR00 Quantikine ELISA Human CXCL1/GRO*α* Immunoassay, R&D Systems).

### 2.3. Statistical Analysis

The statistical analysis was performed using the Statistical Package for the Social Sciences for Windows (SPSS, Version PASW 18.0, Chicago) and R-package software (Version 3.0.2). The distribution of the concentrations of the two measured variables differed from a normal distribution; thus, data was transformed to a logarithmic scale (log 10) for statistical analysis and graphical visualisation (in tables raw values are used). Data with a normal distribution is shown as mean ± standard deviation and was evaluated with the Student *t*-test. Categorical data is expressed as numbers (percentages) and was compared using the *χ* ^2^-test. A *p* value of <0.05 was considered statistically significant. The confidence intervals for the *p* value of <0.05 for multiple statistical analysis were set at the 95% level. For comparison between the two groups (endometriosis patients and controls), the median of the log 10 values with the interquartile ranges is shown in boxplots. The Mann-Whitney-Hugh test was used for the comparison between the two groups and the Kruskal-Wallis test for the comparison of more groups for categorical data.

## 3. Results

### 3.1. Patients with Endometriosis Have Lower BMI Compared to Controls

Before evaluation of the ELISA data, we looked at the clinical characteristics (Table 1) of our patient population and asked whether there is an association of clinical parameters with the disease. Interestingly, BMI differed significantly between endometriosis patients and controls (*p* = 0.002) in our study cohort of 60 Caucasian-origin patients (Table 1). The distribution of the individual endometriosis stages according to the rAFS system is shown in Table 2.  Table 3 shows the mean and median values for sCD40L and CXCL1, as well as pain scores in the endometriosis and control groups. In accordance with previous findings, pain scores in the endometriosis group measured with the VAS were, by trend, higher than in the control group, although they did not reach statistical significance. Therefore, lower BMI and higher pain scores seem to be associated with endometriosis.

### 3.2. The sCD40L and CXCL1 Secretion Is Neither Disease Nor Menstrual Cycle Phase-Dependent

**Figure 1:**
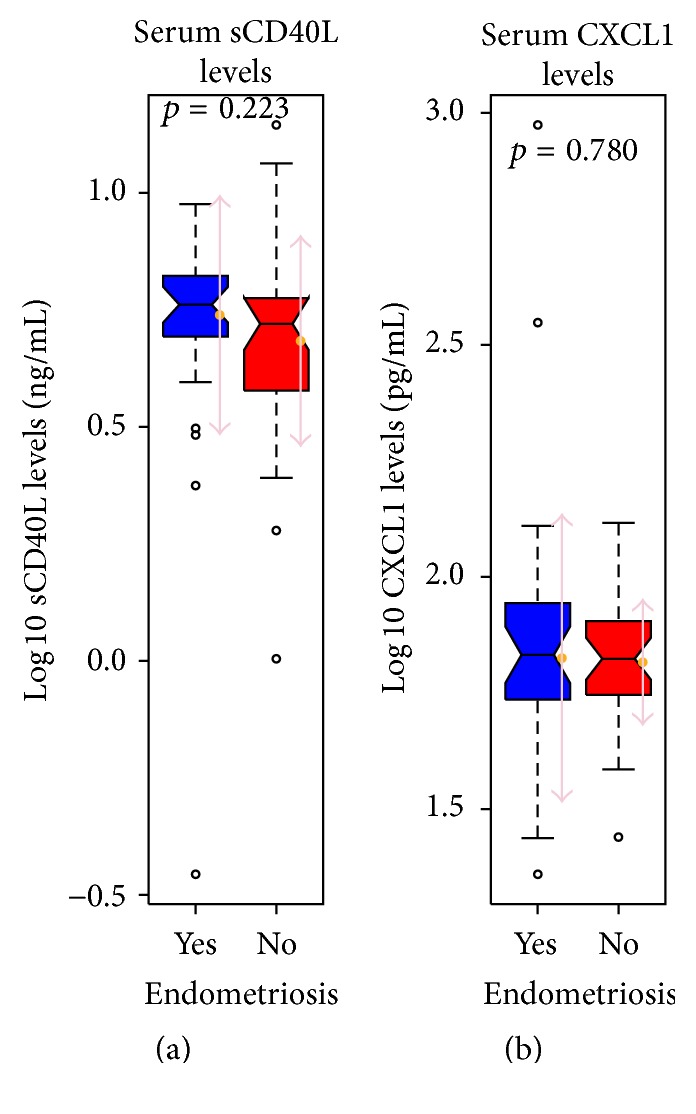
Patients with endometriosis did not show a statistically significant change in the serum levels of both sCD40L and CXCL1. Boxplots showing the comparison between the levels of expression of sCD40L (a) and CXCL1 (b) in serum of patients with endometriosis versus controls. The levels are presented as log 10 and the *p* values are indicated above each plot. Arrows next to the boxplots indicate mean ± standard deviation.

**Figure 2:**
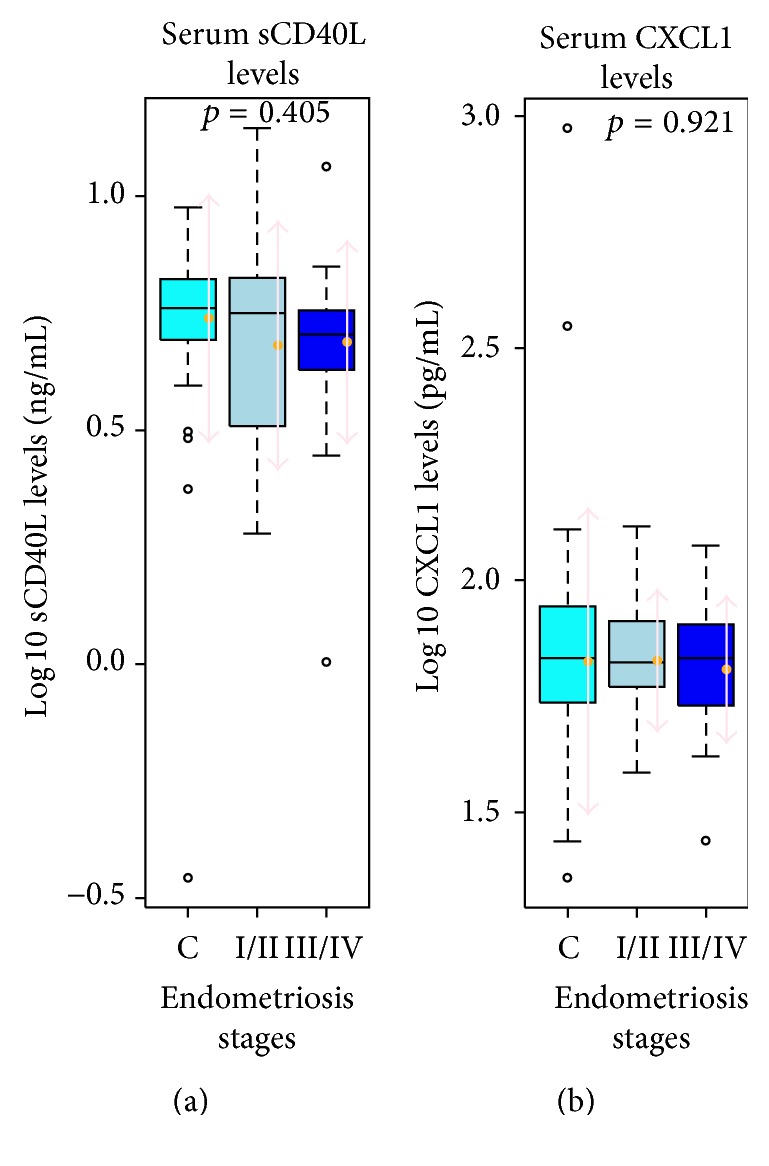
The sCD40L and CXCL1 secretion is disease stage independent. Levels of sCD40L (a) and CXCL1 (b) in the control group and based on the different stages of endometriosis classified by rAFS stage in the endometriosis patients, with no statistically significant differences. C: control group, I/II: endometriosis rAFS minimal-to-mild disease, and III/IV: endometriosis rAFS moderate-to-severe disease.

The Mann-Whitney-Hugh test revealed no statistically significant differences in log 10 sCD40L levels and log 10 CXCL1 levels between endometriosis patients and controls (*p* = 0.223 and *p* = 0.78, resp.) (Figure 1). Nevertheless, there was a slight trend toward a decrease in sCD40L levels in endometriosis patients. The levels of sCD40L and CXCL1 were further compared between controls, minimal-to-mild endometriosis patients (rAFS stage I + II), and moderate-to-severe endometriosis patients (rAFS stage III + IV), which, again, revealed no statistically significant differences between the groups (*p* = 0.405 and *p* = 0.921, resp.) (Figure 2).

**Figure 3:**
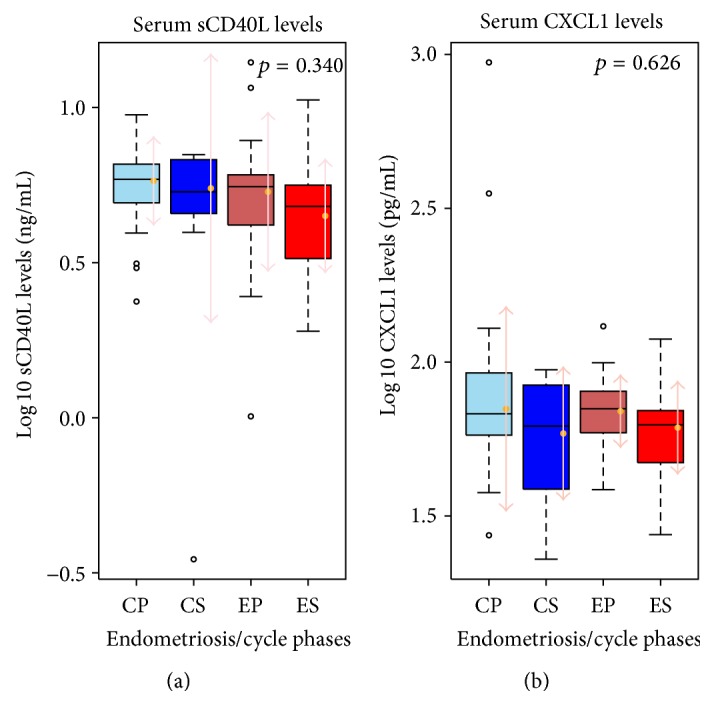
The serum levels of sCD40L and CXCL1 are not differentially regulated during the menstrual cycle in patients with endometriosis versus controls. Boxplots showing levels of sCD40L (a) and CXCL1 (b) among endometriosis patients and controls by menstrual cycle phases, with no statistically significant differences. CP: control proliferative phase, CS: control secretory phase, EP: endometriosis proliferative phase, and ES: endometriosis secretory phase.

To exclude the effect of the different patterns of expression due to differences in cycle phase-dependent regulation of both proteins in controls and endometriosis patients, we analyzed the data after dividing it into subgroups (proliferative versus secretory phase) and searched for cycle phase-specific differences in sCD40L and CXCL1 in controls and endometriosis. Although there seemed to be a trend toward slightly higher levels of both proteins in the proliferative phase compared to the secretory phase of the menstrual cycle in endometriosis patients and in controls (Figure 3), we did not see statistically significant differences associated with the cycle phase (sCD40L, *p* = 0.340 and CXCL1, *p* = 0.626, resp.). This suggests that there is no cycle phase-dependent regulation of sCD40L and CXCL1 secretion.

### 3.3. Patients with Deep Infiltrating Endometriosis Have Lower Serum sCD40L Levels Compared to Controls

**Figure 4:**
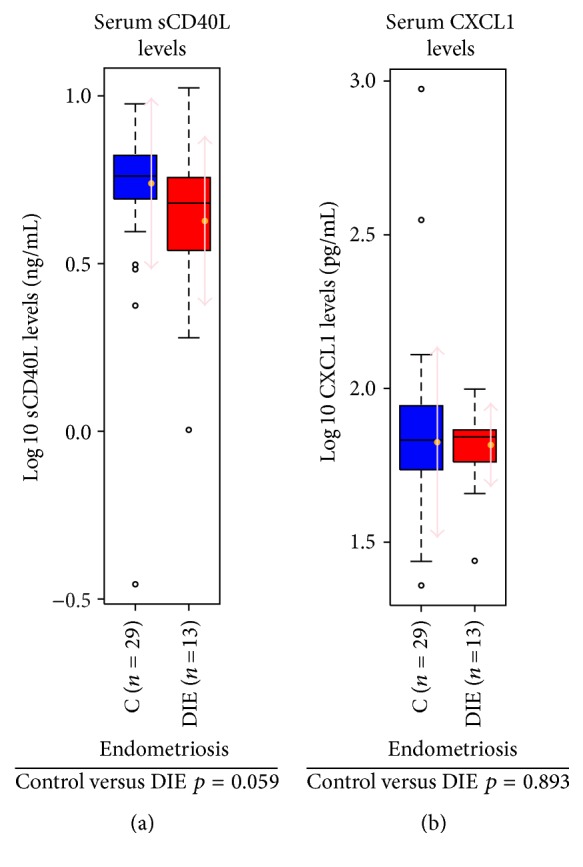
Patients with deep infiltrating endometriosis (DIE) show a tendency of reduced sCD40L levels and no changes in the levels of CXCL1 in serum when compared to controls. Boxplots showing levels of sCD40L (a) and CXCL1 (b) among controls and endometriosis patients with DIE. The number of samples for each group is given in brackets on the *x*-axis and the *p* values of the comparison between the groups are shown below each graph. C: control, DIE: deep infiltrating endometriosis.

As different types of lesion locations might influence the intensity of the inflammatory reaction, we tested whether the levels of sCD40L secretion correlate with the type of the lesion. For this purpose, we divided the endometriosis patients into different groups according to lesion location/type of lesion. A tendency toward a decrease in sCD40L levels in the endometriosis group was seen in accordance with the results of our analysis of all endometriosis patients. In the comparison between controls and endometriosis patients with deep infiltrating endometriosis (*n* = 13), there was a more pronounced difference in the decrease in sCD40L levels between the groups (*p* = 0.059) (Figure 4).

## 4. Discussion

Endometriosis is a disease characterized by the presence of permanent lesions and a continuous inflammatory reaction. Retrograde menstruation is seen as one of the main pathogenetic mechanisms, but as almost 90% of women show patterns of this phenomenon, a proinflammatory microenvironment must be in place in order to promote permanent lesion establishment [6, 27]. In previous studies, it was shown that several inflammatory mediators and proteins involved in angiogenesis are differently expressed between endometriosis patients and controls in the peritoneal fluid and in the tissue itself. Some publications reported differences in the serum values of certain interleukins, cytokines, and some angiogenic factors [28], all supporting the theory of a proinflammatory state that fosters the development and growth of endometriotic lesions [6].

In this study, we could not show statistically significant differences between the controls and endometriosis patients in the levels of the two investigated circulating proteins. Neither did we find significant differences in sCD40L and CXCL1 when looking at patient subgroups with different stages of the disease. Nevertheless, we could confirm the data from the work by Panoulis et al. [21], who could not show differences in the levels of CD40L-protein family in the serum of endometriosis patients compared to controls, using our well-characterized patient cohort. Furthermore, we showed that taking into account the stage of the disease is an important factor in endometriosis studies, which is sometimes neglected. Comparing the two groups of patients, control versus endometriosis patients, we found a trend toward a decrease in sCD40L levels in the endometriosis group, which almost reached the criteria of significance (*p* = 0.059) in the subgroup of patients who suffered from deep infiltrating endometriosis. This suggests a role for sCD40L in more severe cases of endometriosis where the local tissue damage, infiltration, and the initiated inflammatory reaction are more pronounced. As previously shown, CXCL1 is a useful marker in ovarian and cervical cancer and plays a role as a regulator of tumor homeostasis and vascularization and is a good marker for tumor-mediated systemic inflammation [29, 30]. However, our findings argue against the notion that CXCL1 has a function as a circulating regulator of systemic inflammation in endometriosis [5, 24].

It should be noted that, as with all relatively small cohort studies, our findings need to be interpreted carefully, as, in our patient cohort, the significantly different BMI between endometriosis patients and controls could have introduced a bias regarding the levels of circulating cytokine. To date, there is no information in the literature that discusses this possibility. Patients in the endometriosis group showed, overall, a lower BMI, which is in accordance with the data described by others linking endometriosis to a leaner body habitus [31]. In addition, the influence of BMI on the presence of endometriosis, especially in infertile patients [32], might also influence the severity of the inflammatory reaction (locally or systemically) and, consequently, possibly alter the symptoms of the patients. In the PCOS-study from Oktem et al. [20], this factor might have had an important impact on the observed changes in sCD40L levels, as all patients had high BMI values (both controls and endometriosis patients). In our study, the control group had higher BMI values and showed a tendency toward higher levels of sCD40L in the serum. Therefore, a future expanded analysis that includes a larger patient cohort will answer an interesting scientific question about whether the changes in serum sCD40L levels can be linked to advanced stages of endometriosis and whether and how the levels of this particular chemokine correlate with different BMI categories.

## 5. Conclusions

In conclusion, although we could not detect a statistically significant difference in sCD40L and CXCL1 levels between endometriosis patients and controls, this study adds useful clinical data showing the putative relationship between the levels of inflammatory related sCD40L protein and deep infiltrating endometriosis making it a potentially interesting target worth further investigation.

## Figures and Tables

**Table 1:** Patient characteristics.

Baseline characteristics of the endometriosis patients and controls			
Characteristic	Endometriosis, *n* = 31	Controls, *n* = 29	*p* value
Age	34.8 ± 6.9^a^	37.5 ± 6.9^a^	NS

BMI	21.8 ± 4.0^a^	25.6 ± 5.0^a^	.002

Dysmenorrhea	27 (87.1)	17 (58.6)	.013
Mild dysmenorrhea^b^	9 (29)	5 (17.2)	
Moderate-to-severe^b^ dysmenorrhea	18 (58.1)	12 (41.4)	

Dyspareunia	18 (58.1)	12 (41.4)	NS
Mild dyspareunia^b^	9 (29)	2 (6.9)	
Moderate-to-severe^b^ dyspareunia	9 (29)	10 (34.5)	

Cycle phase			
Proliferative	17 (54.8)	21 (72.4)	NS
Secretory	14 (45.2)	8 (27.6)	

*Note*. Values in parentheses represent percentages. NS: not significant.

^a^Values are given in mean ± standard deviation.

^b^Minimal/mild dysmenorrhea/dyspareunia covers VAS from 1 to 5 points, and moderate-to-severe dysmenorrhea/dyspareunia covers VAS from 6 to 10 points.

**Table 2:** Endometriosis patient characteristics. For lesion count, multiple citations are possible.

Endometriosis patient characteristics	
Number of patients (*n*)	*n* = 31
Peritoneal lesions	19 (61.3)
Ovarian lesions	4 (12.9)
Both types	8 (25.8)
Deep infiltrating endometriosis	13 (41.9) of total
rAFS stage	*n*
I	8 (25.8)
II	5 (16.1)
III	12 (38.7)
IV	6 (19.4)

*Note*. Values in parentheses represent percentages.

**Table 3:** Distribution of pain scores measured with the visual analogue scale (VAS), with values from 0 to 10 in endometriosis patients and controls plus serum levels of sCD40L and CXCL1 (numbers represent raw values).

Serum values of sCD40L and CXCL1 and pain scores within groups			
Parameter	Endometriosis	Controls	*p* value
sCD40L^c^	5.38 ± 2.73^a^	5.56 ± 1.83^a^	NS
	5.25 (3.46–5.99)^b^	5.77 (4.93–6.69)^b^	

CXCL1^c^	68.57 ± 21.74^a^	107.96 ± 170.85^a^	NS
	66.65 (53.82–80.70)^b^	67.96 (49.96–90.03)^b^	

Dysmenorrhea	5.68 ± 3.45^a^	4.07 ± 4.11^a^	NS
	7.00 (3.00–8.00)^b^	3.00 (0.00–8.00)^b^	

Dyspareunia	3.13 ± 3.29^a^	2.93 ± 3.75^a^	NS
	3.00 (0.00–7.00)^b^	0.00 (0.00–6.00)^b^	

*Note*. NS: not significant.

^a^Numbers represent mean ± standard deviation.

^b^Numbers represent median and interquartile range.

^c^Concentration of sCD40L is given in ng/mL, and concentration of CXCL1 is given in pg/mL.
